# Scrotal elephantiasis associated with follicular occlusion triad: A case report and literature review

**DOI:** 10.1097/MD.0000000000015263

**Published:** 2019-04-19

**Authors:** Jing-Chao Liu, Xi-Gao Liu, Chao Xu, Hai-Feng Zhao, Xian-Zhou Jiang

**Affiliations:** Department of Urology, Qilu Hospital of Shandong University, Jinan, China.

**Keywords:** case report, follicular occlusion triad, hidradenitis suppurativa, scrotal elephantiasis, urologic diseases

## Abstract

**Rationale:**

Follicular occlusion triad (FOT) is an autosomal recessive inherited disease and no more than 3 variants of the triad have been reported. We give a report in which scrotal elephantiasis is a variant of FOT and further perform a literature review.

**Patient concerns:**

A 41-year-old man came to us because of a large scrotal cyst and generalized skin lesions that had occurred over the past 10 years. The generalized skin lesions consisted of hidradenitis suppurativa on the perineum and back, acne conglobata in the armpit, and dissecting cellulitis of the scalp. He took antibiotics for a long time but achieved poor effect. Furthermore, he told his father and elder brother also manifested such skin lesions.

**Diagnoses:**

Magnetic resonance showed a mass in the left scrotum with clear boundaries. A routine blood test showed a high leukocyte level of 12 × 10^9^/L and a hemoglobin content of 78 g/L. C-reactive-protein increased. Series of autoimmune antibody tests were negative. The postoperative pathologic findings showed that the mass was an epidermoid cyst, and hematoxylin and eosin staining showed hyperkeratosis of the skin as well as inflammatory and edematous changes. A diagnosis of a variant of FOT was made.

**Interventions:**

We removed skin abscesses and lesioned the inner part with hydrogen peroxide. Then we performed an excision of the scrotal lesion.

**Outcome:**

The patient recovered well and had no evidence of recurrence at a 16-month follow-up.

**Lessons:**

We reported a case in which scrotal elephantiasis was a variant of FOT and surgical intervention played an important role in secondary urologic diseases.

## 1 Introduction

Urologic diseases have a close relationship with follicular occlusion triad (FOT). FOT is a rare disease that manifests as 3 symptoms that occur in a single organism at the same time, including hidradenitis suppurativa (HS), acne conglobata (AC), and dissecting cellulitis of the scalp.^[1]^ FOT is an autosomal recessive inherited disease. FOT can frequently involve inguinogenital areas, and it may lead to various kinds of urologic diseases, such as scrotal elephantiasis, urinary leakage, and penis disfigurement. Unfortunately, there are no effective therapeutic methods for FOT, and the mechanisms of secondary urologic diseases are also unclear. To identify the relationship between FOT and urologic disease, we report a case in which scrotal elephantiasis is a variant of FOT; additionally, we reviewed the related literature.

## 2 Case presentation

### 2.1 Ethic approval

Our case study was approved by Qilu Hospital Review Board and was accordant with Helsinki II declaration. The patient also gave written informed consent for publication.

### 2.2 Case report

**Figure 1:**
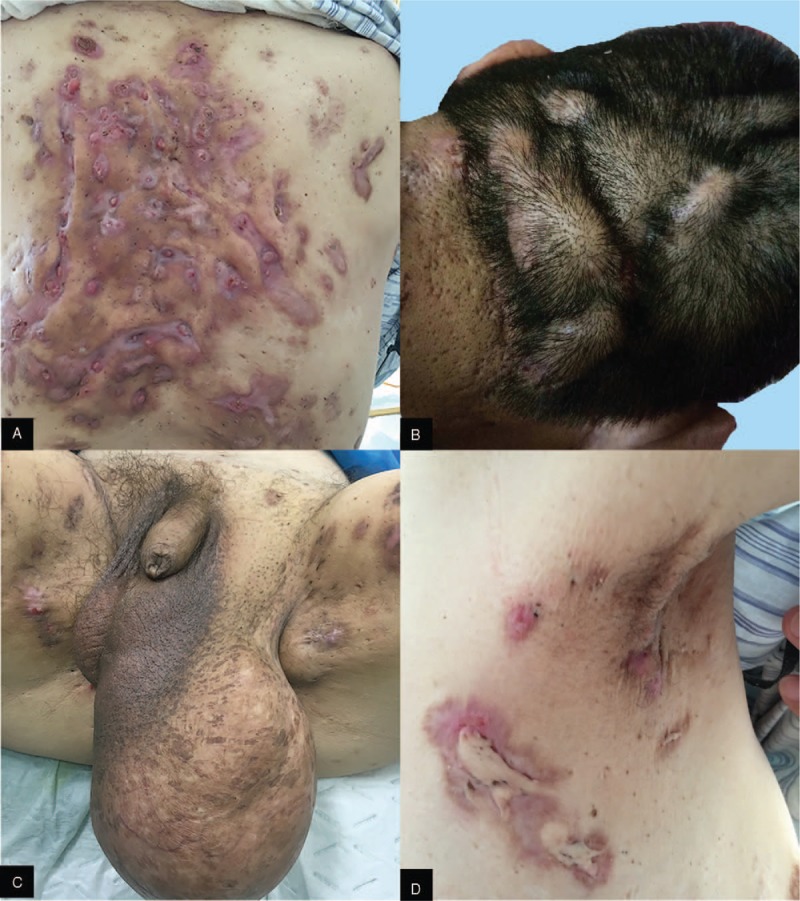
(A) Hidradenitis suppurativa on the patient's back. (B) Dissecting cellulitis of the scalp. (C) Large scrotal cyst and hidradenitis suppurativa in the perineum. (D) Acne conglobata and hidradenitis suppurativa presentation in his armpit.

A 41-year-old man came to us because of a large scrotal cyst and generalized skin lesions that had occurred over the past 10 years. The generalized skin lesions consisted of HS on the perineum and back (Fig. 1A, C), AC in the armpit (Fig. 1D), and dissecting cellulitis of the scalp (Fig. 1B). His left scrotal cyst reached a profound size of 20 cm × 13 cm × 9 cm (Fig. 1C). He initially presented generalized skin lesions and then scrotal cyst developed 1 year later. Magnetic resonance showed a mass in the left scrotum with clear boundaries. The right testis measured 3.1 cm × 3.1 cm × 1.9 cm, and the left testis measured 3.1 cm × 2.8 cm × 1.9 cm. The morphology and envelope of the bilateral testes were normal. He took antibiotics orally and intermittently to prevent infection, but this approach achieved a poor effect. In addition, he had a history of type 2 diabetes and denied any history of promiscuity. A routine blood test showed a high leukocyte level of 12 × 10^9^/L and a hemoglobin content of 78 g/L. C-reactive-protein increased. We also performed a series of autoimmune antibody tests, and all the results were negative. He refused any genetic test.

**Figure 2:**
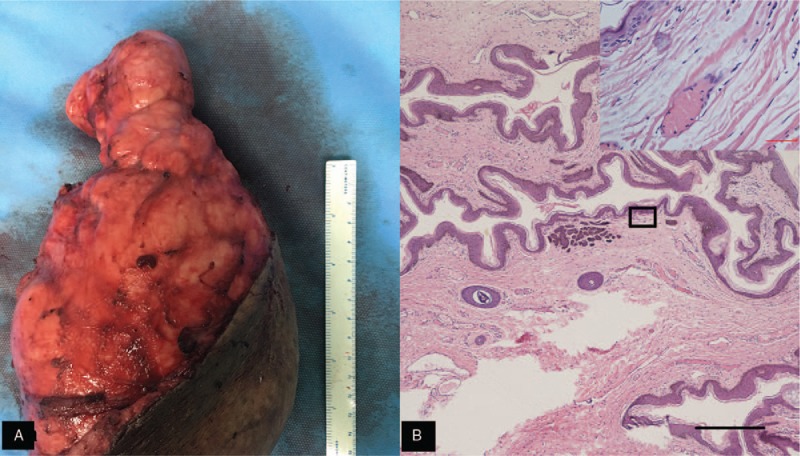
(A) Postoperatively, pathologic changes in the cyst. (B) Hematoxylin and eosin (H&E) staining showed hyperkeratosis and edematous changes in the skin. Scale bars = 400 μm.

Based on the clinical and laboratory findings, a diagnosis of a variant of FOT was made. We removed skin abscesses and washed the inner part with hydrogen peroxide and saline. We covered the wounds with vacuum suction and performed an excision of the scrotal lesion. The boundaries of the large cyst were in close proximity with the tunica vaginalis of the left scrotum. We separated the large cyst entity from the tunica vaginalis carefully, without any damage to the testis. The postoperative pathologic findings showed that the mass was an epidermoid cyst, and hematoxylin and eosin (H&E) staining showed hyperkeratosis of the skin as well as inflammatory and edematous changes (Fig. 2A, B). Until now (16 months later), there has been no evidence of any recurrence, and the patient is recovering well. The patient has provided informed consent for publication of the case.

## 3 Discussion

The FOT was documented in a single patient by Chicarilli in 1987.^[1]^ The lesion process initially manifests as hair follicle obstruction, giving rise to deep folliculitis, abscess, partial sinus, and scarring of the skin. Boer and Weltevreden pointed out the pilosebaceous unit was the primary occlusion site and that any apocrine ductal occlusion was secondary, and any site containing follicles could be affected.^[2]^ There were several reports of scrotal elephantiasis with longstanding HS.^[3]^ To our knowledge, there have been no reports of scrotal elephantiasis with FOT. Over the past few years, several cases of some variants of FOT have been reported.^[4,5]^ Gandhi et al presented a case of FOT associated with reticulate pigmentary disorder in 2013.^[4]^ Coincidentally, the man in our case had a similar symptom of reticulate pigmentary disorder in his perineum and buttocks. Meyers et al presented a rare case with an extensive cluster of exophytic nodules that developed on his chin, which was also a variant of FOT.^[5]^ We believe the difference between his and our case lies in the distribution region of FOT. There were a large number of sebaceous glands in the scrotum and chin, leading to a high prevalence of FOT. Several professionals pointed out that the presence of bacterial infection was secondary.^[6,7]^ We performed several microbial cultivations from skin lesions and from the scrotal cyst during the patient's hospitalization. There were almost no pathogenic bacteria cultivated at the start of treatment. We believed this lack of bacteria was because of the short-term disease course and the active treatments adopted.

Worldwide, there are almost no effective therapeutic methods for FOT. Early diagnosis and timely surgical treatment play key roles in the triad. Many clinical experts have shared their treatment experiences in published papers.^[8,9]^ But there remain no effective drugs. In consideration of our case, antibiotics, isotretinoin, and finasteride were ineffective for the patient; we had to give him a treatment with oral zinc, and we achieved optimistic feedback. This outcome supported Kobayashi's view of the oral zinc therapy.^[9]^ We performed a simple debridement to avoid secondary infection and an excision of the scrotal lesion to thoroughly resolve the patient's discomfort. Many experts thought of the triad as a kind of autosomal recessive inherited disease. Wang et al pointed out that γ-secretase gene mutation was the main mechanism, and their findings were published in SCIENCE^[10]^ (http://fanyi.baidu.com/?aldtype=16047 - zh/en/javascript:void(0)). Thus therapeutic methods at the molecular genetic level should be further researched in the future.

Scrotal elephantiasis has not been previously reported to occur with FOT but has always occurred with HS. We retrospectively reviewed the English literature concerning urogenital diseases and HS in Medline, PubMed, and Web of Science, yielding 9 reported cases. These reviewed literatures may broaden our horizon for treatment of such scrotal elephantiasis in our case. It is because that such scrotal elephantiasis in our case may be due to longstanding HS. On the contrary, disorders of FOT (HS, AC, and dissecting cellulitis of the scalp) posed similar pathogenic and histologic features.^[5]^ Corresponding urogenital diseases may have similar mechanism both in the reviewed cases above and our cases. Each patient's treatment and prognosis are summarized in Table 1.^[3,11–17]^ Timely surgical intervention is necessary to achieve good prognosis for secondary urogenital diseases.

**Table 1:**
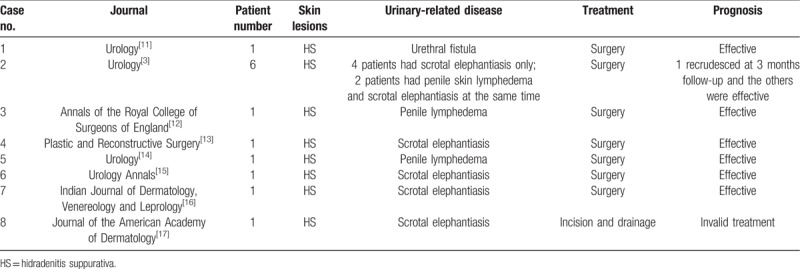
Clinical features in eight cases.

In conclusion, we gave a report in which scrotal elephantiasis was a variant of FOT. Surgical intervention plays an important role in secondary urogenital diseases, and therapeutic methods at the molecular genetic level should be researched in the future.

## Author contributions

XZJ carried out the design and revision of this study. HFZ and CX took part in the operation and collected clinical data. JCL collected pathological information. JCL and XGL drafted the manuscript. All authors have read and approved the final version of the manuscript, and agreed with the order of presentation of the authors.

**Conceptualization:** Xian-Zhou Jiang.

**Data curation:** Jing-Chao Liu, Chao Xu, Hai-Feng Zhao.

**Formal analysis:** Hai-Feng Zhao.

**Investigation:** Hai-Feng Zhao.

**Project administration:** Xian-Zhou Jiang.

**Writing – original draft:** Xi-Gao Liu, Jing-Chao Liu.

**Writing – review & editing:** Jing-Chao Liu.
